# Food insecurity and cultural food access among international college students in the USA

**DOI:** 10.1017/S136898002510178X

**Published:** 2026-01-06

**Authors:** Na Zuo, Angela Jungbluth, Katherine E. Speirs

**Affiliations:** 1 Department of Agricultural and Applied Economics, College of Agriculture, Life and Environmental Sciences, University of Arizona, 1064 E. Lowell Street, N384, Tucson, AZ 85719, USA; 2 Department of Agricultural Economics, Ferguson College of Agriculture, Oklahoma State University, 311 North Monroe Street, Stillwater, OK 74078, USA; 3 Human Development and Family Science, The Norton School of Human Ecology, University of Arizona, 650 North Park Avenue, Tucson, AZ 85721, USA

**Keywords:** International college students, Food insecurity, Cultural food access, Cultural food affordability

## Abstract

**Objective:**

A high rate of food insecurity among college students has been documented in various studies. Knowledge gaps exist regarding food insecurity and cultural food access among international college students. We explored the demographic correlations of food insecurity and cultural food access and affordability for international college students.

**Design:**

Cross-sectional online survey from 2 to 16 November 2022.

**Setting:**

A public university in the southwestern USA.

**Participants:**

Three hundred and thirty-five international undergraduate and graduate students.

**Results:**

About 22 % of the sample reported high food security, 18 % marginal food security, 30 % low food security and 31 % very low food security. Twenty-seven percent reported that they were able to find cultural foods at the university, and 29 % reported that they were able to afford the cultural foods available on campus. Enrolment status, primary caregiver status, housing location and vehicle ownership predicted food security status. Region of origin, gender, being a primary caregiver for an adult with special needs and vehicle ownership were associated with access to cultural foods. Region of origin, being a primary caregiver for children, housing location and vehicle ownership were associated with being able to afford cultural foods on campus. The predictors differ between undergraduate and graduate international students.

**Conclusion:**

Researchers and student services professionals who develop programmes and resources to support international students should consider differences within the group of international students, especially differences by region of origin and degree status (undergraduate *v*. graduate), and work to ensure students have access to reliable transportation.

High rates of food insecurity among college students are well-documented^(e.g. 1, 2)^ and concerning, given the links between food insecurity and poor physical and mental health and academic performance^(3–8)^. However, little attention has been paid specifically to international college students in the USA.

Defined as students who are not citizens of the country where they are attending school, international students are an important segment of the US college student population. During the 2022–2023 academic year, about 1·1 million international students enrolled in postsecondary institutions in the USA, representing 4·2 % of the total enrolment^(9)^, with the largest percentage (71 %) coming from Asian countries^(10)^.

There is some evidence that high rates of international college students in the USA report food insecurity^(11)^. In a sample of 2,654 students collected at a minority and Hispanic-serving US institution in April 2020, 39 % of international students reported experiencing food insecurity^(12)^. Evidence also shows that international students are more likely to experience food insecurity than US citizens. Soldavini, Berner and Da Silva found that international student status increased the odds of experiencing food insecurity *v*. food security for undergraduate students and marginal food security *v*. high food security for graduate students in a sample of 4,819 students collected in 2016 at a large US public university^(13)^.

Higher rates of food insecurity among international students may be partially explained by the unique financial challenges they face. In the USA, international students are not allowed to receive federal financial aid, which includes grants and work-study positions where aid does not need to be repaid, as well as federal loans that often have more favourable terms than private loans^(14,15)^. In the USA, international students are also not allowed to receive Supplemental Nutrition Assistance Program benefits, which are an important food resource that provides near-cash benefits that can be used to purchase food at many food retailers^(16,17)^.

To effectively design programmes and resources to ensure food security, it is important to understand the demographic correlations of food insecurity for international students. We know of only two studies that examine demographic or descriptive correlations of food insecurity for international students in the USA. Soldavini, Andrew and Berner conducted an online survey with a convenience sample of 263 international undergraduate and graduate students at a southeastern US university in fall 2016^(18)^. Twenty-five percent of their sample reported experiencing food insecurity. In examining the relationship between sixteen demographic variables and food insecurity, they found that gender, year in school, possession of a car, self-reported physical health and frequency of cooking were statistically significantly associated with food insecurity. Using a smaller sample (*n* 73) collected in 2021, Ibiyemi, Najam and Oldewage-Theron found that out of seven descriptive variables, only length of time in the USA was associated with food insecurity^(19)^. International students who had been in the USA for less than a year were statistically significantly more likely to be food insecure.

Access to cultural foods is important to consider when thinking about how best to provide food-related resources for international students. Cultural foods are those foods that international students eat in their home countries. Access to these foods can promote well-being, feelings of belonging, cultural identity development and maintenance and can alleviate acculturative stress^(20,21)^. However, there is evidence that international students in the USA struggle to access cultural foods^(22,23)^.

The present study aims to add to the literature on international college students’ experiences of food insecurity and accessing cultural foods in order to aid efforts to support international students. We address two research questions. First, what are the demographic correlates of food insecurity for international college students? Second, what are the demographic correlates of cultural food accessibility and affordability for international college students? We examined the demographic correlates of food insecurity and access to cultural foods for undergraduates and graduate students separately, given evidence from the literature that undergraduate and graduate students are two distinct groups^(18,23)^. Graduate students seem to have lower rates of food insecurity than undergraduates^(22,24)^, and there are different demographic correlates of food insecurity for these two groups of students^(18)^.

## Methods

### Study design and participants

An online survey was conducted in November 2022, at a large public land-grant university located about 60 miles from the Mexican border in a town with just over 500,000 people. Invitations to participate in the study were sent to all international undergraduate and graduate students who were enrolled at the university’s main campus in fall 2022. International students were defined as enrolled students who were not citizens of the USA, US permanent residents, refugees, asylees or Jay Treaty status holders. In 2022–2023, international students made up 6·8 % of the total enrolment in the study university^(25)^. Of the 3,460 international students, 335 completed the survey, a 9·7 % response rate.

### Measures

Food insecurity was measured using the six-item USDA Household Food Security Survey Module with a 30-day reference period^(26)^. We coded responses into four food security statuses: high food security, marginal food security, low food security and very low food security. We distinguished marginal food security from high food security, as increasing evidence suggests that even marginal food security is linked to adverse outcomes comparable to low or very low food security for college students^(8,27)^.

Cultural food accessibility was measured using the question: ‘To what degree do you agree or disagree with the following statement: I am able to find food at the (university) similar to what I am used to eating at home’ (strongly disagree, disagree, unsure/no opinion, agree and strongly agree). Cultural food affordability was measured using the question: ‘To what degree do you agree or disagree with the following statement: I can afford the food that I am culturally accustomed to on the (university) campus’ (strongly disagree, disagree, unsure/no opinion, agree and strongly agree). The following definition of cultural foods, which is based on the definition in the US Centers for Disease Control and Prevention Food Service Guideline Toolkit^(28)^, was provided in the survey: ‘Cultural foods, also called traditional dishes or culturally preferred foods, is used to describe safe and nutritious foods that are based on the consumer’s cultural identity and represent the traditions, beliefs and practices of a geographic region, ethnic group, religious body or cross-cultural community’. The questions used to measure cultural food accessibility and affordability were validated with expert review and pilot testing. Subject matter experts, such as the director of the campus pantry, reviewed the questions and provided feedback. We also pilot tested the survey in a college class with thirty-six undergraduate students, including international students, at the same university where the data were collected. These students took the survey and provided feedback about the survey design and questions in interviews with the study authors.

We measured thirteen demographic characteristics: gender (woman, man, other; participants in the other category selected either ‘prefer not to answer’ or ‘non-binary’), first-generation status (no parents or guardians who earned a bachelor’s degree or higher), primary caregiver for child(ren) under the age of 18, primary caregiver for an adult with special care needs, employment status (employed *v*. not employed but looking for work or not employed and not looking for work), meal plan (yes *v*. no or not sure), off-campus housing (on campus (university housing, fraternity or sorority house), off campus, and no stable residence), vehicle ownership (participants were coded as owning a vehicle if they reported using their own vehicle in the past 30 days to get food or do other activities) and, on average, the percent of food consumed that is cultural foods. Participants were also asked to report which country they were a citizen of. We used the world location list^(29)^ to categorise respondents into four regions: Africa, Asia, Europe and Canada, and Latin America and the Caribbean. We created a Europe and Canada category because only two Canadians were in the North America category. Age, degree (undergraduate or graduate student) and enrolment status (full time or part time) were merged into our dataset from institutional data. All data were collected anonymously.

### Analyses

Frequencies and percentages were used to describe the sample. Fisher’s exact tests were performed to assess statistical differences between undergraduate and graduate students on the demographic characteristics and dependent variables (food security status, cultural food accessibility and cultural food affordability). We used ordered probit models with robust standard errors to explore associations between demographic characteristics and the three dependent variables. We fit three models for undergraduates (one for each dependent variable) and three for graduate students, for a total of six models. The lowest level of statistical significance was accepted as *P* < 0·05.

## Results

### Participant characteristics

We were able to compare our sample to the full population of international students on some of our study variables. Our sample closely matches the population of international students at the university on full-time enrolment (82 % in our sample and in the population of international students) and off-campus residence (84 % in our sample and 88 % in the population) and overrepresents graduate students (66 % in our sample *v*. 45 % in the population), first-generation students (31 % *v*. 16 %) and women (51 % *v*. 21 %).

Full sample descriptives are presented in Table 1. Undergraduate and graduate students in our sample were significantly different on age, enrolment status, region of origin, first-generation status, being a caregiver for children, employment status, whether they have a meal plan, housing location and the amount of cultural food in their diet.

**Table 1.** Sample descriptives (*n* 335)

	All students (*n* 335)		Undergraduate students (*n* 112)		Graduate students (*n* 223)		
	*n*	%	*n*	%	*n*	%	Fisher’s exact test *P*-value
Age							< 0·001
< 18 years old	5	1·5	5	4·5	0	0·0	
18–22 years old	97	29·0	90	80·4	7	3·1	
> 22 years old	235	69·6	17	15·2	216	96·9	
Enrolled part time^*^	59	17·6	2	1·8	57	25·6	< 0·001
Region of origin							0·0275
Africa	26	7·8	11	9·8	15	6·7	
Asia	237	70·7	67	59·8	170	76·2	
Europe and Canada	26	7·8	12	10·7	14	6·3	
Latin America and the Caribbean	46	13·7	22	19·6	24	10·8	
Gender							0·9957
Woman	170	50·7	57	50·9	113	50·7	
Man	151	45·1	51	45·5	100	44·8	
Other^†^	7	2·1	2	1·8	5	2·2	
First-generation student	104	31·0	26	23·2	78	35·0	0·0335
Primary caregiver for child(ren)	17	5·1	1	0·9	16	7·2	0·015
Primary caregiver for adult(s)	6	1·8	1	0·9	5	2·2	2·5285
Employed	218	65·1	47	42·0	171	76·7	< 0·001
Meal plan	29	8·7	22	19·6	7	3·1	< 0·001
Housing							
On-campus (university, fraternity or sorority houses)	54	16·1	29	25·9	25	11·2	0·001
Off-campus housing	279	83·3	83	74·1	196	87·9	
No stable residence	2	0·6	0	0·0	2	0·9	
Vehicle Ownership	95	28·4	25	22·3	70	31·4	0·0748
Percent of foods eaten that are, on average, cultural foods							0·0224
0 %	11	3·3	6	5·4	5	2·2	
1–25 %	73	21·8	30	26·8	43	19·3	
26–50 %	83	24·8	30	26·8	53	23·8	
51–75 %	89	26·6	31	27·7	58	26·0	
76–99 %	74	22·1	14	12·5	60	26·9	
100 %	5	1·5	1	0·9	4	1·8	
Food security status							0·5068
Very low food security	105	31·3	36	32·1	69	30·9	
Low food security	99	29·6	38	33·9	61	27·4	
Marginal food security	59	17·6	17	15·2	42	18·8	
High food security	72	21·5	21	18·8	51	22·9	
I am able to find foods at the (university) similar to what I am used to eating at home.							0·6578
Strongly disagree	75	22·5	28	25·0	47	21·2	
Disagree	128	38·3	40	35·7	88	39·6	
Unsure/no opinion	40	12·0	16	14·3	24	10·8	
Agree	78	23·4	23	20·5	55	24·8	
Strongly agree	13	3·9	5	4·5	8	3·6	
I can afford the food that I am culturally accustomed to on the (university) campus.							0·5863
Strongly disagree	55	16·4	18	16·1	37	16·6	
Disagree	115	34·3	34	30·4	81	36·3	
Unsure/no opinion	69	20·6	29	25·9	40	17·9	
Agree	83	24·8	27	24·1	56	25·1	
Strongly agree	13	3·9	4	3·6	9	4·0	

* The sample size for enrolment status was 333.

† The ‘other’ category for gender includes two participants who selected ‘non-binary’ and five who selected ‘prefer not to answer’.

About 22 % of the sample reported high food security, 18 %marginal food security, 30 % low food security and 31 % very low food security. Half of the sample reported that over 50 % of their diet was cultural foods, and only 3 % reported eating, on average, no cultural foods. About a quarter reported that they were able to find cultural foods at the university and were able to afford the cultural foods available on campus.

### Demographic correlations of food insecurity

Reporting lower levels of food security was statistically significantly associated with part-time enrolment, region of origin being Latin America and the Caribbean, primary caregiver for child(ren) and not having a vehicle for international undergraduate students, being a primary caregiver for adult(s), not having a stable residence and not having a vehicle for international graduate students (Table 2, columns 1 and 4).

**Table 2.** Ordered probit regression results for key explanatory variables related to food security level, cultural food accessibility and cultural food affordability

	Undergraduate students				Graduate students		
		(1)	(2)	(3)	(4)	(5)	(6)
		Food security^†^	Cultural food accessibility^‡^	Cultural food affordability^§^	Food security^†^	Cultural food accessibility^‡^	Cultural food affordability^§^
Age							
18–22 years old (reference category)							
>22 years old	Coefficient	–0·517	–0·910	0·230	0·029	0·816	–0·880
	se	0·568	0·538	0·493	1·018	0·498	0·940
<18 years old	Coefficient	1·237	1·059	0·862			
	se	0·805	1·479	0·672			
Enrolled part time	Coefficient	–15·162***	1·126	0·706	–0·263	0·360	0·320
	se	0·896	0·999	1·921	0·294	0·325	0·307
Region of origin							
Africa	Coefficient	0·093	–2·393**	–0·421	–0·007	–0·154	1·749***
	se	0·723	0·950	0·773	0·900	0·577	0·651
Asia	Coefficient	–0·044	–1·495**	–0·111	0·678	0·980**	1·194**
	se	0·597	0·762	0·595	0·485	0·397	0·507
Europe and Canada (reference category)							
Latin America and the Caribbean	Coefficient	–1·244**	–3·025***	–0·949	0·662	0·924	0·552
	se	0·601	0·726	0·629	0·543	0·513	0·678
Gender							
Woman (reference category)							
Man	Coefficient	–0·737	–0·737	–0·191	0·124	0·359	0·290
	se	0·407	0·407	0·437	0·267	0·273	0·281
Other	Coefficient	1·161	1·161	–0·392	–0·232	–1·584**	–1·191
	se	0·953	0·953	0·548	0·628	0·701	0·608
First-generation student	Coefficient	0·037	–0·035	0·505	–0·269	0·472	–0·216
	se	0·449	0·416	0·470	0·243	0·271	0·267
Primary caregiver for child(ren)	Coefficient	–1·720**	1·414	–15·051***	0·336	–0·109	–0·955**
	se	0·867	1·504	1·280	0·488	0·498	0·429
Primary caregiver for adult(s)	Coefficient	0·825	–15·715***	0·052	–1·245**	–2·060	0·637
	se	1·029	1·605	0·829	0·570	1·218	0·894
Employed	Coefficient	–0·568	–0·686	–0·181	0·511	–0·248	–0·224
	se	0·474	0·438	0·425	0·332	0·276	0·308
Meal plan	Coefficient	–0·356	–0·548	–0·307	0·890	0·293	1·149
	se	0·514	0·648	0·593	0·588	0·707	0·660
Housing							
On-campus (reference category)							
Off-campus	Coefficient	–0·132	0·344	–1·186**	–0·346	–0·266	–0·211
	se	0·482	0·499	0·568	0·310	0·443	0·365
No stable residence	Coefficient				–1·847**	–1·173	–0·980
	se				0·909	1·691	0·610
Vehicle ownership	Coefficient	1·644***	1·125**	1·010**	0·696**	–0·447	0·605**
	se	0·446	0·438	0·445	0·307	0·303	0·306
*n*		112	112	112	222	221	222
Pseudo-*R* ^2^		0·108	0·127	0·056	0·032	0·046	0·047
Log-likelihood-value		–132·957	–142·356	–155·099	–294·124	–295·338	–306·834

** *P* < 0·05, ***P < 0·01.

† Food security was coded 1 = very low food security, 2 = low food security, 3 = marginal food security, 4 = high food security.

‡ Cultural food accessibility was coded 1 = strongly disagree with the statement ‘I am able to find food at the (university) similar to what I am used to eating at home’, 2 = disagree with the statement, 3 = unsure or no opinion about the statement, 4 = agree with the statement, 5 = strongly agree with the statement.

§ Cultural food affordability was coded 1 = strongly disagree with the statement ‘I can afford the food that I am culturally accustomed to on the (university) campus’, 2 = disagree with the statement, 3 = unsure or no opinion about the statement, 4 = agree with the statement, 5 = strongly agree with the statement.

### Demographic correlations of cultural food accessibility and affordability

Reporting a lack of cultural food accessibility was associated with region of origin, being a caregiver for an adult and not owning a vehicle for undergraduate students. For graduate students, region of origin and identifying with a gender other than man or woman were associated with cultural food inaccessibility (Table 2, columns 2 and 5). Not being able to afford cultural foods on campus was associated with being a caregiver for children, living off campus and not owning a vehicle for undergraduate students. For graduate students, region of origin, being a caregiver for children and not owning a vehicle were associated with cultural food unaffordability (Table 2, columns 3 and 6).

## Discussion

Sixty-one percent of our sample of international students at a large land-grant university reported low or very low food security, which is higher than estimates from other studies of international students, that is, between 32 and 39 %^(11,12,18,19)^. Although all of these estimates were made using non-representative samples from one university, taken together, they suggest that international students experience high rates of food insecurity and universities should provide resources specifically for international students, especially given these students’ lack of access to federal food resources.

Our results concerning the demographic correlates of food insecurity, cultural food accessibility and affordability suggest the importance of access to a vehicle that can be used to purchase food. Vehicle ownership was associated with food security status and cultural food accessibility for undergraduate students and cultural food affordability for both undergraduate and graduate students. Soldavini, Andrew and Berner (2022) and Glick, Winham and Shelly (2025) found similar positive associations between having a car and food security in samples of international students^(18,22)^. Students have also reported having to travel off campus to find culturally appropriate foods^(30)^. Glick and colleagues also found that international students were more likely than US-born white students to report lacking reliable transportation^(22)^. Owning a car is likely important because it allows students to purchase food off campus, where they can often access less expensive food^(31)^ and more authentic cultural foods. However, vehicle ownership might not be realistic for all or even many international students. Other solutions could include making sure food pantries on campus stock international foods as there is some evidence that international students are more likely to use campus pantries than US college students^(11,22)^ and lobbying for increased public transportation or providing university shuttles from campus to parts of the surrounding communities that have grocery stores or restaurants where cultural foods can be purchased.

Our results also suggest that the region of the world from which a student comes to the USA shapes their experiences. Region of origin was associated with food security status and cultural food accessibility for undergraduate students and cultural food accessibility and affordability for graduate students. Differences in food security status and access to cultural foods likely vary by region of origin due to both characteristics of the individual students (e.g. culinary literacy skills, knowledge and beliefs about food resources on or near campus, social support, experiences of discrimination) and the university and local community environment (e.g. the availability of holistic supports for basic and academic needs on campus; dining halls, restaurants and grocery stores selling cultural or inexpensive foods on or near campus; availability of cultural foods through food resources)^(32,33)^. Although this exploratory study is not designed to make definitive statements about which regions of origin may predict food insecurity or lack of access to cultural foods, our findings suggest that it is likely important for researchers and student services professionals to consider differences within international students based on region of origin, rather than considering international students as one homogeneous group.

Additionally, fitting separate models for undergraduate and graduate students revealed differences between these groups. These findings, along with several studies showing that class year is related to food security status,^(e.g. 18, 23)^ suggest that undergraduate and graduate students should be considered separately both when conducting research and designing support programmes.

Our findings should be considered in light of study limitations. The use of a self-selected sample from one university’s international student population limits the generalisability of our findings. Our sample included only a small number of students in some groups (e.g. caregivers for children or adults with special needs and non-binary or gender non-conforming students), making it hard to draw conclusions about these groups of international students. In the future, researchers should oversample these groups in order to address questions about them. The 9·7 % response rate to our survey introduced non-response bias, although it is similar to response rates found in other studies using a similar census sampling approach for the entire university, which also report response rates below 11 %^(8,18)^ and a study of international students with a 4% response rate^(19)^. Despite these limitations, our study adds to the literature by exploring food insecurity using a four-category (rather than three- or two-category) measure of food security status for a sample of international college students, an important but often ignored group, and widening the focus to include access to and affordability of cultural foods.

## References

[1] Bruening M , Argo K , Payne-Sturges D et al. (2017) The struggle is real: a systematic review of food insecurity on postsecondary education campuses. J Acad Nutr Diet 117, 1767–1791. 10.1016/j.jand.2017.05.022 28754200 PMC6901286

[2] Nikolaus CJ , An R , Ellison B et al. (2020) Food insecurity among college students in the United States: a scoping review. Adv Nutr 11, 327–348. 10.1093/advances/nmz111 31644787 PMC7442331

[3] Hagedorn RL , Olfert MD , MacNell L et al. (2021) College student sleep quality and mental and physical health are associated with food insecurity in a multi-campus study. Public Health Nutr 24, 4305–4312. 10.1017/S1368980021001191 33745495 PMC8385605

[4] Kim Y & Murphy J (2023) Mental health, food insecurity, and economic hardship among college students during the COVID-19 pandemic. Health Soc Work 48, 124–132. 10.1093/hsw/hlad006 36898047

[5] Loofbourrow BM & Scherr RE (2023) Food insecurity in higher education: a contemporary review of impacts and explorations of solutions. Int J Environ Res Public Health 20, 5884. 10.3390/ijerph20105884 37239614 PMC10217872

[6] Raskind IG , Haardörfer R & Berg CJ (2019) Food insecurity, psychosocial health and academic performance among college and university students in Georgia, USA. Public Health Nutr 22, 476–485. 10.1017/S1368980018003439 30724722 PMC6366643

[7] Weaver RR , Vaughn NA , Hendricks SP et al. (2020) University student food insecurity and academic performance. J Am Coll Health 68, 727–733. 10.1080/07448481.2019.1600522 31063031

[8] Coakley KE , Cargas S , Walsh-Dilley M et al. (2022) Basic needs insecurities are associated with anxiety, depression, and poor health among university students in the State of New Mexico. J Community Health 47, 454–463. 10.1007/s10900-022-01073-9 35124789 PMC8818275

[9] U.S. Department of Education, National Center for Education Statistics, Integrated Postsecondary Education Data System (IPEDS) (n.d.) 12-Month Enrollment Component 2022–23 Provisional Data. https://nces.ed.gov/ipeds/trendgenerator/app/build-table/2/2?rid=65&cid=1 (accessed January 2026).

[10] National Center for Education Statistics (2024) Table 310.20. Foreign Students Enrolled in Postsecondary Institutions in the United States, by Continent, Region, and Selected Countries of Origin: Selected Academic Years, 1980–81 through 2022–23. https://nces.ed.gov/programs/digest/d23/tables/dt23_310.20.asp (accessed January 2026).

[11] El Zein A , Mathews AE , House L et al. (2018) Why are hungry college students not seeking help? Predictors of and barriers to using an on-campus food pantry. Nutrients 10, 1163. 10.3390/nu10091163 30149599 PMC6163327

[12] Mechler H , Coakley K , Walsh-Dilley M et al. (2024) Examining the relationship between food insecurity and academic performance: implications for diversity and equity in higher education. J Coll Stud Retent Res Theory Pract 26, 3–18. 10.1177/15210251211053863

[13] Soldavini J , Berner M & Da Silva J (2019) Rates of and characteristics associated with food insecurity differ among undergraduate and graduate students at a large public university in the Southeast United States. Prev Med Rep 14, 100836. 10.1016/j.pmedr.2019.100836 30886818 PMC6403080

[14] Federal Student Aid (n.d.) When it Comes to Paying for College, Career School, or Graduate School, Federal Student Loans Can Offer Several Advantages Over Private Student Loans. United States Department of Education. https://studentaid.gov/understand-aid/types/loans/federal-vs-private (accessed January 2026).

[15] US Department of Education (n.d.) Eligibility for Non-U.S. Citizens. https://studentaid.gov/understand-aid/eligibility/requirements/non-us-citizens (accessed January 2026).

[16] Freudenberg N , Goldrick-Rab S & Poppendieck J (2019) College students and SNAP: the new face of food insecurity in the United States. Am J Public Health 109, 1652–1658. 31622149 10.2105/AJPH.2019.305332 PMC6836795

[17] US Department of Agriculture (2024) SNAP Eligibility for Non-Citizens. https://www.fns.usda.gov/snap/recipient/eligibility/non-citizen (accessed April 2025).

[18] Soldavini J , Andrew H & Berner M (2022) Campus-based food insecurity: the case of international students at a Southeastern University. J Stud Aff Res Pract 59, 338–351. 10.1080/19496591.2021.1997755

[19] Ibiyemi T , Najam W & Oldewage-Theron W (2024) Hungry, stressed, and away from ‘home’: predictors of food security and perceived stress among international students. Am J Health Promot 38, 1238–1242. 10.1177/08901171241257092 38832401

[20] Rodyna SN (2023) We are very proud and very tempted and determined to make this food. Arbutus Rev 14, 81–96. 10.18357/tar141202321371

[21] Wright KE , Lucero JE , Ferguson JK et al. (2021) The influence of cultural food security on cultural identity and well-being: a qualitative comparison between second-generation American and international students in the United States. Ecol Food Nutr 60, 636–662. 10.1080/03670244.2021.1875455 33632041

[22] Glick AA , Winham DM & Shelley MC (2025) Food insecurity predictors differ for white, multicultural, and international college students in the United States. Nutrients 17, 237. 39861367 10.3390/nu17020237 PMC11767435

[23] Goldman BJ , Freiria CN , Landry MJ et al. (2024) Research trends and gaps concerning food insecurity in college students in the United States: a scoping review. J Am Coll Health 1–40. 10.1080/07448481.2024.2351420 38870038

[24] Abbey EL , Brown M & Karpinski C (2022) Prevalence of food insecurity in the general college population and student-athletes: a review of the literature. Curr Nutr Rep 11, 185–205. 10.1007/s13668-022-00394-4 35218475 PMC8881554

[25] The University of Arizona, University Analytics & Institutional Research (2025) Enrollment. https://uair.arizona.edu/content/enrollment (accessed January 2026).

[26] US Department of Agriculture, Economic Research Service (2024) U.S. Household Food Security Survey Module: Six-Item Short Form. https://www.ers.usda.gov/topics/food-nutrition-assistance/food-security-in-the-us/survey-tools#six (accessed January 2026).

[27] Dana LM , Wright J , Ward R et al. (2023) Food insecurity, food assistance, and psychological distress among university students: cross-sectional survey Western Australia, 2020. Nutrients 15, 2431. 10.3390/nu15112431 37299396 PMC10255323

[28] Centers for Disease Control and Prevention (2024) Consider Cultural Food Preferences: FAQ. FSG Toolkit. https://www.cdc.gov/food-service-guidelines-toolkit/php/strategize-act/cultural-food-preferences.html (accessed January 2026).

[29] United Nations, Department of Economic and Social Affairs, Population Division (2022) World Population Prospects 2022, Online Edition. https://www.un.org/development/desa/pd/sites/www.un.org.development.desa.pd/files/wpp2022_summary_of_results.pdf(accessed January 2026).

[30] Watson TD , Malan H , Glik D et al. (2017) College students identify university support for basic needs and life skills as key ingredient in addressing food insecurity on campus. Calif Agr 71, 130–138. 10.3733/ca.2017a0023

[31] Zigmont VA , Anziano J , Schwartz E et al. (2023) Captive market pricing and lack of transportation: a survey of undergraduate food insecurity at a public university in New England. Am J Health Promot 37, 313–323. 36112939 10.1177/08901171221127006

[32] Brown NI , Buro AW , Jones R et al. (2023) Multi-level determinants of food insecurity among racially and ethnically diverse college students. Nutrients 15, 4065. 10.3390/nu15184065 37764847 PMC10535142

[33] DeBate R , Jarvis JE , Perez J et al. (2025) Socio-ecological determinants of food pantry use among food insecure racial and ethnic diverse college students. Am J Lifestyle Med. Published online: 10 Jun 2025. doi: 10.1177/15598276251351585. PMC12158980 40521229

